# A Method to Quantify Mean Hypertension Treatment Daily Dose Intensity Using Health Care System Data

**DOI:** 10.1001/jamanetworkopen.2020.34059

**Published:** 2021-01-15

**Authors:** Lillian Min, Jin-Kyung Ha, Carole E. Aubert, Timothy P. Hofer, Jeremy B. Sussman, Kenneth M. Langa, Mary Tinetti, Hyungjin Myra Kim, Matthew L. Maciejewski, Leah Gillon, Angela Larkin, Chiao-Li Chan, Eve A. Kerr, Dawn Bravata, William C. Cushman

**Affiliations:** 1 Veterans Affairs Geriatric Research, Education, and Clinical Center, Veterans Affairs Ann Arbor Medical Center, Ann Arbor, Michigan; 2 Division of Geriatric and Palliative Medicine, Department of Medicine, University of Michigan, Ann Arbor; 3 Veterans Affairs Center for Clinical Management Research, Health Services Research and Development Center of Innovation, Ann Arbor, Michigan; 4 Institute of Healthcare Policy and Innovation, University of Michigan, Ann Arbor; 5 Department of General Internal Medicine, Bern University Hospital, University of Bern, Bern, Switzerland; 6 Institute of Primary Healthcare, University of Bern, Bern, Switzerland; 7 Division of General Internal Medicine, Department of Internal Medicine, University of Michigan, Ann Arbor; 8 Institute for Social Research, University of Michigan, Ann Arbor; 9 Section of Geriatrics, Department of Medicine, Yale School of Medicine, New Haven, Connecticut; 10 Consulting for Statistics, Computing & Analytics Research, University of Michigan, Ann Arbor; 11 Department of Biostatistics, University of Michigan Medical School, Ann Arbor; 12 Center of Innovation to Accelerate Discovery and Practice Transformation, Veterans Affairs Healthcare System, Durham, North Carolina; 13 Department of Population Health Sciences, Duke University, Durham, North Carolina; 14 Veterans Affairs Health Services Research and Development Center for Health Information and Communication, Richard L. Roudebush Veterans Affairs Medical Center, Indianapolis, Indiana; 15 Department of Medicine, Indiana University School of Medicine, Indianapolis; 16 Department of Neurology, Indiana University School of Medicine, Indianapolis; 17 Center for Health Services Research, Regenstrief Institute, Indianapolis, Indiana; 18 Department of Preventive Medicine, University of Tennessee Health Science Center, Memphis; 19 Medical Service, Memphis Veterans Affairs Medical Center, Memphis, Tennessee

## Abstract

**Importance:**

Simple measures of hypertension treatment, such as achievement of blood pressure (BP) targets, ignore the intensity of treatment once the BP target is met. High-intensity treatment involves increased treatment burden and can be associated with potential adverse effects in older adults. A method was previously developed to identify older patients receiving intense hypertension treatment by low BP and number of BP medications using national Veterans Health Administration and Medicare Part D administrative pharmacy data to evaluate which BP medications a patient is likely taking on any given day.

**Objective:**

To further develop and validate a method to more precisely quantify dose intensity of hypertension treatment using only health system administrative pharmacy fill data.

**Design, Setting, and Participants:**

Observational, cross-sectional study of 319 randomly selected older veterans in the national Veterans Health Administration health care system who were taking multiple BP-lowering medications and had a total of 3625 ambulatory care visits from July 1, 2011, to June 30, 2013. Measure development and medical record review occurred January 1, 2017, through November 30, 2018, and data analysis was conducted from December 1, 2019, to August 31, 2020.

**Main Outcomes and Measures:**

For each BP-lowering medication, a moderate hypertension daily dose (HDD) was defined as half the maximum dose above which no further clinical benefit has been demonstrated by that medication in hypertension trials. Patients’ total HDD was calculated using pharmacy data (pharmacy HDDs), accounting for substantial delays in refills (>30 days) when a patient’s pill supply was stretched (eg, cutting existing pills in half). As an external comparison, the pharmacy HDDs were correlated with doses manually extracted from clinicians’ visit notes (clinically noted HDDs). How well the pharmacy HDDs correlated with clinically noted HDDs was calculated (using C statistics). To facilitate interpretation, HDDs were described in association with the number of medications.

**Results:**

A total of 316 patients (99.1%) were male; the mean (SD) age was 75.6 (7.2) years. Pharmacy HDDs were highly correlated (*r* = 0.92) with clinically noted HDDs, with a mean (SD) of 2.7 (1.8) for pharmacy HDDs and 2.8 (1.8) for clinically noted HDDs. Pharmacy HDDs correlated with high-intensity, clinically noted HDDs ranging from a C statistic of 92.8% (95% CI, 92.0%-93.7%) for 2 or more clinically noted HDDs to 88.1% (95% CI, 85.5%-90.6%) for 6 or more clinically noted HDDs.

**Conclusions and Relevance:**

This study suggests that health system pharmacy data may be used to accurately quantify hypertension regimen dose intensity. Together with clinic-measured BP, this tool can be used in future health system–based research or quality improvement efforts to fine-tune, manage, and optimize hypertension treatment in older adults.

## Key Points

**Question:**

Can the intensity of hypertension treatment by total dose burden for older patients be accurately quantified using only health system administrative pharmacy fill data?

**Findings:**

In this cross-sectional study, dose intensity measured using pharmacy data in a national health care system was highly correlated with dose intensity documented in clinical notes by using a new algorithm that can be applied to an individual patient on any given day.

**Meaning:**

This study suggests that intensity of hypertension treatment can be measured using pharmacy data at a more detailed level than using medication counts or blood pressure alone for quality comparison or as a research tool.

## Teaser

This cross-sectional study develops and validates a method to more precisely quantify dose intensity of hypertension treatment using only health system administrative pharmacy fill data.

## Introduction

The current approach to assess the quality of hypertension treatment in health care systems remains limited to dichotomous measures of blood pressure (BP) (ie, percentage of patients with a BP controlled below target) regardless of the recommended number and doses of medications. However, there is increasing evidence that intensifying treatment is associated with clinical benefit for older adults, either by increasing doses and starting new agents^1,2,3,4,5^ or by prescribing specific medication classes for their noncardiovascular benefits (eg, angiotensin-converting enzyme inhibitors for kidney protection).^6^ As our health care system responds by developing new guidelines and measures to promote lower BP goals, we need more refined tools to guide safe approaches to treatment intensification for older adults.

National public health efforts track hypertension treatment by surveying whether patients are receiving pharmacologic treatment or not,^7,8^ while hypertension trials demonstrate effectiveness as the increment in the number of BP medications.^2,5,9^ However, neither of these approaches captures dose intensity, a component of medical treatment that is of particular interest to older adults, who are prone to dose-related adverse effects as a result of frailty, total medication burden, or comorbid conditions and who often prefer lower medication intensity.^10,11^

Being able to estimate a standardized hypertension daily dose (HDD) across medications of differing potency may enable health care systems to develop approaches to intensifying treatment in addition to monitoring the BP level achieved. Moreover, a decrease in dose intensity may be an older patient’s only indicator of improved adherence to nonpharmacologic hypertension treatment (eg, diet)^12^ because patients might be able to reduce their medication doses while maintaining appropriate BP control. Last, such a tool would also more precisely identify which older patients might be appropriate candidates for deintensification of treatment.

An algorithm was previously developed and validated to assess treatment intensity measured as the number of medications based only on reliable administrative pharmacy fill data within a complex electronic medical record and health care data system.^13^ The goal of the present study was to (1) extend the method to capture daily dose, defined as moderate HDD, for each BP-lowering medication; (2) validate that the total HDDs calculated from pharmacy data across multiple medications reflect the intensity of patients’ overall antihypertension medication regimens; and (3) describe the association between the new HDD measure and the traditional medication count.

## Methods

### Population

A previous study described the sampling strategy for this cohort of moderately to intensely treated veterans^13^ selected from a national sample of older (≥65 years) Veterans Health Administration patients with hypertension (*International Classification of Diseases, Ninth Revision* code 401.x), receiving primary care over 2 years (from July 1, 2011, to June 30, 2013), taking 3 or more BP medications, and having a systolic BP less than 120 mm Hg for 2 or more consecutive visits. The strategy ensured that we had an adequate number of visits while receiving multiple BP medications to validate. Of 66 412 older veterans meeting the inclusion criteria, we randomly selected 319 patients with 3625 primary care visits.^13^ This study was approved by the Veterans Affairs (VA) institutional review board with a waiver of informed consent granted to obtain an unbiased, retrospective sample among eligible patients (many who were not expected to be alive at the time the study was conducted). We followed the Strengthening the Reporting of Observational Studies in Epidemiology (STROBE) reporting guideline.

### Assignment of Standardized Moderate Doses

We (L.M. and W.C.C.) reviewed all antihypertensive medications on the VA national formulary,^14^ in the American College of Cardiology/American Heart Association hypertension guidelines,^15^ and in the Anatomic Therapeutic Class/Defined Daily Dose (ATC/DDD) database.^16^ For each medication, we identified the maximum dose demonstrated in controlled hypertension trials above which no further clinical benefit was expected as supported by evidence synthesis in the seventh and eighth reports of the Joint National Committee^17,18^ and the American College of Cardiology/American Heart Association hypertension guidelines^15^ and supplemented by specific literature reviews.^19,20,21,22,23,24,25,26,27,28^ These documents also identified minimum starting doses for older patients (Table 1).^15,17,18,19,20,21,22,24,26,27,28^ Last, we defined a standardized unit of measurement, the HDD, as half the maximum beneficial dose because most of the antihypertensive effectiveness is achieved at moderate doses.^29^ For example, lisinopril’s maximum effective dose is 40 mg, so we chose 20 mg as 1 standardized HDD unit. Thus, a patient with 2 HDDs could be taking a maximum dose of 1 medication or half the maximum beneficial dose of 2 different BP medications.

**Table 1.** Blood Pressure Medications and Doses

Class (No.) and medication	Geriatric starting dose, mg/d	Dose corresponding to 1 HDD, mg/d	Maximum dose based on reference, mg/d	Source
Angiotensin agents (1)				
Benazepril hydrochloride	10	20	40	2017 ACC/AHA^15^ and JNC 7^17^
Captopril	25	50	100	JNC 7^17^
Enalapril maleate	5	20	40	2017 ACC/AHA^15^ and JNC 7^17^
Fosinopril sodium	10	20	40	2017 ACC/AHA^15^ and JNC 7^17^
Lisinopril	10	20	40	2017 ACC/AHA,^15^ JNC 7,^17^ and JNC 8^18^
Moexipril hydrochloride	7.5	15	30	2017 ACC/AHA^15^ and JNC 7^17^
Perindopril erbumine	4	8	16	2017 ACC/AHA^15^
Quinapril hydrochloride	20	40	80	2017 ACC/AHA^15^ and JNC 7^17^
Ramipril	2.5	10	20	2017 ACC/AHA^15^ and JNC 7^17^
Trandolapril	1	2	4	2017 ACC/AHA^15^ and JNC 7^17^
Azilsartan medoxemil	20	40	80	2017 ACC/AHA^15^
Candesartan cilexetil	8	16	32	2017 ACC/AHA^15^ and JNC 7^17^
Eprosartan mesylate	400	600	800	JNC 7^17^ and JNC 8^18^
Irbesartan	75	150	300	2017 ACC/AHA^15^
Losartan potassium	25	50	100	JNC 7^17^
Olmesartan medoxomil	5	20	40	2017 ACC/AHA^15^ and JNC 7^17^
Telmisartan	20	40	80	2017 ACC/AHA^15^ and JNC 7^17^
Valsartan	80	160	320	2017 ACC/AHA,^15^ JNC 7,^17^ and JNC 8^18^
Calcium channel blockers (2)				
Amlodipine besylate	2.5	5	10	2017 ACC/AHA^15^ and JNC 7^17^
Diltiazem hydrochloride	120	240	420	2017 ACC/AHA^15^ and JNC 7^17^
Felodipine	2.5	5	10	2017 ACC/AHA^15^
Isradipine	2.5	5	10	JNC 7^17^
Nicardipine hydrochloride	60	90	120	2017 ACC/AHA^15^ and JNC 7^17^
Nifedipine	30	60	90^a^	2017 ACC/AHA^15^
Nisoldipine	8.5	17	34	2017 ACC/AHA^15^
Verapamil hydrochloride	120	240	480	JNC 7^17^
Thiazide and thiazide-like diuretics (3)				
Chlorthalidone	12.5	12.5	25	2017 ACC/AHA,^15^ JNC 7,^17^ and JNC 8^18^
Hydrochlorothiazide	12.5	25	50	JNC 7^17^ and JNC 8^18^
Indapamide	1.25	2.5	5	Chaffman et al^20^ and Caruso et al^19^
Metolazone	2.5	5	5^a^	2017 ACC/AHA^15^
Polythiazide	2	2	4	JNC 7^17^
Bendroflumethiazide	2.5	5	10	JNC 8^18^
Potassium-sparing diuretics (4)				
Amiloride hydrochloride	2.5	5	10	2017 ACC/AHA^15^ and JNC 7^17^
Eplerenone	25	50	100	2017 ACC/AHA^15^ and JNC 7^17^
Spironolactone	25	50	100	2017 ACC/AHA^15^
Triamterene	25	50	100	2017 ACC/AHA^15^ and JNC 7^17^
β-Blockers (5)				
Acebutolol hydrochloride	200	400	800	2017 ACC/AHA^15^ and JNC 7^17^
Atenolol	25	50	100	2017 ACC/AHA,^15^ JNC 7,^17^ and JNC 8^18^
Bisoprolol fumarate	2.5	5	10	2017 ACC/AHA^15^ and JNC 7^17^
Carvedilol phosphate	12.5	25	50	2017 ACC/AHA^15^ and JNC 7^17^
Labetalol hydrochloride	200	400	800	2017 ACC/AHA^15^ and JNC 7^17^
Metoprolol tartrate or succinate	50	100	200	2017 ACC/AHA,^15^ JNC 7,^17^ and JNC 8^18^
Nadolol	40	80	120	2017 ACC/AHA^15^ and JNC 7^17^
Nebivolol hydrochloride	5	20	40	2017 ACC/AHA^15^
Penbutolol sulfate	10	20	40	2017 ACC/AHA^15^ and JNC 7^17^
Pindolol	10	30	60	2017 ACC/AHA^15^
Propranolol hydrochloride	40	80	160	2017 ACC/AHA^15^
Sotalol hydrochloride^b^	160	320	640	Sundquist et al^27^
Centrally acting sympathetic agonist (6)				
Clonidine hydrochloride (oral; patch)	0.2; 0.1	0.4; 0.2	0.8; 0.3	2017 ACC/AHA^15^ and JNC 7^17^
Guanfacine hydrochloride	0.5	1	2	2017 ACC/AHA^15^ and JNC 7^17^
Methyldopa	250	500	1000	2017 ACC/AHA^15^ and JNC 7^17^
Reserpine	0.05	0.125	0.25	JNC 7^17^
Vasodilators (7)				
Hydralazine hydrochloride	50	100	200	JNC 7^17^
Minoxidil	5	20	80	2017 ACC/AHA^15^ and JNC 7^17^
Direct renin blocker (8)				
Aliskiren hemifumarate	75	150	300	2017 ACC/AHA^15^
α-Blockers (9)^b^				
Doxazosin mesylate^b^	4	8	16	Kirby^22^
Prazosin hydrochloride^b^	2	10	20	Levy^24^
Silodosin^b^	4	4	8	Yoshida et al^28^
Terazosin hydrochloride^b^	2	10	20	2017 ACC/AHA^15^ and JNC 7^17^
Loop diuretics (10)^b^				
Bumetanide^b^	0.5	1	2	JNC 7^17^
Furosemide^b^	20	40	80	Musini et al^26^
Torsemide^b^	2.5	5	10	2017 ACC/AHA^15^ and JNC 7^17^
Nitrates (11)^b^				
Isosorbide dinitrate (oral; patch)^b^	30; 4.8	120; 9.6	480; 19.2	Duchier et al^21^
Isosorbide mononitrate^b^	30	60	240	Duchier et al^21^

Abbreviations: ACC/AHA, American College of Cardiology/American Heart Association; HDD, hypertension daily dose; JNC, Joint National Committee.

^a^ The original maximal beneficial dose reflects the revised guidelines for hypertension by the ACC/AHA, which were updated only for these 2 medications in 2018.^15^

^b^ Minimum and maximum beneficial doses and citations are provided for these blood pressure–lowering medications that can be used in the treatment of hypertension as second-line agents; however, these medications were not included in the medical record validation analysis owing to frequent as-needed dosing, frequent changes in dosing between visits and refills, and inconsistent documentation in the clinical notes as being part of the treatment plan for essential hypertension.

We assigned HDDs for (1) angiotensin-converting enzyme inhibitors or angiotensin receptor blockers, (2) calcium channel blockers, (3) thiazides or thiazide-like diuretics, (4) potassium-sparing diuretics, (5) β-blockers, (6) centrally acting α-agonists, (7) vasodilators, and (8) direct renin blockers (Table 1). Loop diuretics, nitrates, and α-blockers also decrease BP, so we assigned HDDs to these agents. However, these classes were excluded from analysis because they were frequently prescribed on an as-needed basis, with dose changes between visits for nonhypertension care, or were rarely documented as part of hypertension treatment.

The HDDs that we assigned were updated from the ATC/DDDs to reflect modern hypertension trial evidence, as trials have used new classes and newer doses of medications not historically used for hypertension control. Whereas the explicit purpose of the ATC is to establish a stable international standardized dose unit,^30^ the clinical evidence led us to assign HDDs that differed from the DDDs in 32 of 55 medications (58.2%) that we reviewed. Hypertension daily doses were lower than the DDDs for several β-blockers (eg, metoprolol tartrate: HDD, 100 mg; DDD, 150 mg) and diuretics (eg, spironolactone: HDD, 50 mg; DDD, 75 mg), whereas the HDDs were greater than the DDDs for angiotensin-converting enzyme inhibitors and angiotensin receptor blockers (eg, lisinopril: HDD, 20 mg; DDD, 10 mg) (eTable 1 in the Supplement).

### Data

We obtained medication data from the national VA Clinical Data Warehouse, including Veterans Health Administration pharmacy records and Medicare Part D claims during the study period. From the claims, we extracted medication name, doses, date of fill, and days’ supply of pills dispensed. Data analysis occurred at the level of the visit. The total HDD was calculated on the date of every eligible visit. Eligible visits included outpatient primary care visits (with physicians, nurses, advanced practice clinicians, and pharmacists) in family, general, and geriatric medicine as well as outpatient nephrology, endocrinology, cardiology, and neurology owing to their expertise in managing BP medications.

### Base Algorithm to Evaluate Medication Exposure During Each Visit

A previous study has described how pharmacy fills can be used to evaluate which BP medications a veteran is receiving on any given day.^13^ In brief, our approach sorts all pharmacy fills as a longitudinal series of events, classifying BP medications into classes. Second, using the pattern of fill dates and pill supply in association with the visit day, the algorithm evaluates whether or not a patient is taking a medication on a long-term basis. Medications need to be filled within 186 days both before and after a visit to be considered as a continuous medication. For discontinued medications (filled prior to but not after the visit), the visit was required to fall within 80% of days’ supply after starting a new medication or within 90% of days’ supply of dispensing an old medication.^13^ This algorithm evaluated the number of medications.

### Determining Total Standardized Doses of BP Medications

Next, we assigned standardized dose units to each medication at each visit, modifying a method to capture refill compliance.^31^ First, for each medication, we captured doses dispensed at the last fill prior to the visit (“prefill”). Then, we evaluated the time between the prefill and the refill. For example, if 90 pills of lisinopril, 40 mg, were dispensed for a 90-day supply (3600 mg in total) and the refill happened within 90 days, then we assigned 3600 mg/90 days or 40 mg/d to lisinopril. Thus, 40 mg/d, or 2 HDD units, for lisinopril would be added to the total HDDs. If the time elapsed between the prefill and the refill was greater than the days’ supply and the visit occurred after the expected refill date, we presumed that the daily dose was stretched uniformly across the days. For example, if 120 days elapsed, the daily dose was considered to be 30 mg (3600 mg/120 days) instead of 40 mg, or 1.5 standardized HDD units. For discontinued medications, we assumed the daily dose to be the last dispensed daily dose. The methods, including SAS codes, are described in further detail in eTable 2 in the Supplement. The total HDD was calculated for each medication for every visit.

### Clinical Note Review

To validate HDD estimates from pharmacy data only (or pharmacy HDDs), 4 trained abstractors (L.G. and A.L.) reviewed free-text clinical notes in the medical record (including narrative, medication list, problem list, assessment, and plan) to obtain all documented BP medications and doses during the study period. To evaluate the BP medications taken on the day of each eligible visit (ie, prior to any recommended changes), reviewers read all visit notes, including interval notes (ie, telephone notes, emergency department visits, hospital discharge summaries, and non-VA facility visits). Any medication documented as being present but of unknown dose was presumed to be 1 HDD, which occurred in 0.2% of medications abstracted (22 of 9488). We calculated HDDs by the record (clinically noted HDDs) as a simple sum of HDDs across all medications in each visit note. In contrast to pharmacy HDDs, we made no adjustments to the clinically noted HDDs for late refills.

### Statistical Analysis

Statistical analysis was performed from December 1, 2019, to August 31, 2020. The correlation coefficient between pharmacy HDDs and clinically noted HDDs was the primary basis for assessing concordance, accounting for the clustering of visits for each patient. With the clinically noted HDDs as the external standard, we tested the ability for pharmacy HDDs to correlate with clinically noted HDDs across varying criteria for a more intense regimen (≥2, ≥3, ≥4, ≥5, and ≥6 HDDs) using sensitivity, specificity, and overall C statistic.

Finally, to help us understand the clinical meaning of the pharmacy HDDs, we described the distribution (mean, range, SD, and mode) of the number of BP medications corresponding to pharmacy HDD categories. We highlighted the visits in which using simple counts of medications might potentially overestimate intensity if used alone (a number greater than HDDs by 0.5 units) and in which the number might potentially underestimate intensity if used alone (a number less than HDDs by 2 units). Data were analyzed using SAS statistical software, version 9.4 (SAS Institute Inc) and Stata 15 (StataCorp).

## Results

The sample of 319 patients with 3625 visits (mean [SD], 11.4 [8.3] visits per patient) was 99.1% male (n = 316) with a mean (SD) age of 75.6 (7.2) years. The mean (SD) systolic BP was 121.9 (16.4) mm Hg (range, 79.8-201.5 mm Hg) (Table 2). The mean (SD) number of BP medications according to the medical record was 2.6 (1.0). The mean (SD) clinically noted HDD was 2.8 (1.8) (range, 0-11), similar to the mean (SD) pharmacy HDD (2.7 [1.8]; range, 0-11) (ie, between 2 and 3 medications at a moderate dose). Among the first-line medications, the mean (SD) class-specific HDD unit ranged from 0.9 (0.5) for thiazides to 1.3 (0.6) for calcium channel blockers. The correlation between pharmacy HDDs and clinically noted HDDs at the level of the visit (3625 visits, adjusted for 319 patient clusters) was 0.92 (Figure). After the addition of the Medicare Part D data, the correlation coefficient did not change. The pharmacy HDDs were lower than clinically noted HDDs in 1209 visits (33.4%), higher than clinically noted HDDs in 553 visits (15.3%), and identical to clinically noted HDDs in approximately half the visits (1863 [51.4%]).

**Table 2.** Sample Characteristics^a^^,^^b^

Variable	Mean (SD) [range]
SBP per visit, mm Hg	121.9 (16.4) [79.8-201.5]
Taking ≥3 medications, No. (%)	1890 (52.1)
HDD	2.8 (1.8) [0-11]^c^
On ≥3 HDDs, No. (%)	1585 (43.7)
Medication class (any during study period), No. (%)	
β-Blocker	2912 (80.3)
Angiotensin-converting enzyme inhibitor or angiotensin receptor blocker	2856 (78.8)
Calcium channel blocker	1518 (41.9)
Thiazide or thiazide-like diuretic	1195 (33.0)
Potassium-sparing diuretic	583 (16.1)
Other vasodilator	140 (3.9)
Centrally acting α-blocker	117 (3.2)
Direct renin blocker	24 (0.7)
HDD according to medication class	
β-Blocker	1.0 (0.8) [0.125-4]
Angiotensin-converting enzyme inhibitor or angiotensin receptor blocker	1.2 (0.8) [0.125-6]
Calcium channel blocker	1.3 (0.6) [0.125-4]
Thiazide-like diuretic	0.9 (0.5) [0.072-2]
Potassium-sparing diuretic	0.6 (0.3) [0.25-2.75]
Other vasodilator	0.9 (0.6) [0.1-3]
Centrally acting α-blocker	0.6 (0.4) [0.125-1.5]
Direct renin blocker	1.3 (0.5) [1-2]

Abbreviations: HDD, hypertension daily dose; SBP, systolic blood pressure.

^a^ Total of 3625 visits for 319 patients.

^b^ All HDD and medication counts are from clinical note review.

^c^ All patients had at least 3 medications on 2 consecutive visits over 2 years, but this did not preclude some having individual visits while taking fewer than 3 medications. Of 3625 visits, 50 visits had 0 HDDs, thus resulting in the range of HDDs including 0.

**Figure.**
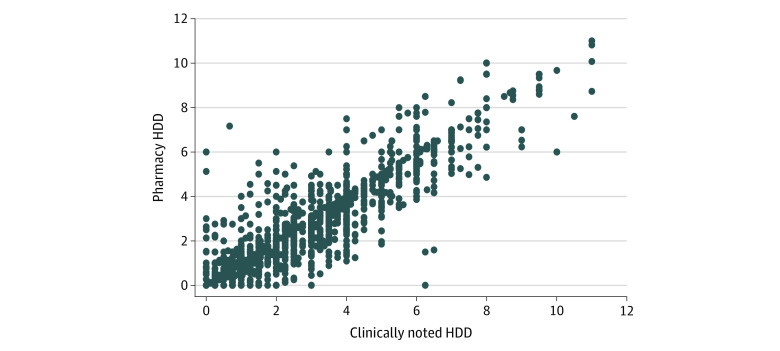
Correlation Between Pharmacy and Clinically Noted Hypertension Daily Doses (HDDs) Coefficient of correlation *r* = 0.92 (95% CI, 0.89-0.95); *P* < .001; *R*^2^ = 0.85. Adjusted for clustering of visits within patient.

The ability of the pharmacy HDD to detect a clinically noted HDD regimen of 2 or more, 3 or more, 4 or more, 5 or more, or 6 or more HDDs was the most sensitive (91.5%) for 2 or more HDDs and the most specific (99.0%) for 6 or more HDDs (Table 3). Considering the overall ability of pharmacy HDDs to correlate with clinically noted HDDs, C statistics ranged from 92.8% (95% CI, 92.0%-93.7%) for 2 or more clinically noted HDDs to 88.1% (95% CI, 85.5%-90.6%) for 6 or more clinically noted HDDs.

**Table 3.** Pharmacy HDD Discrimination for More Intensive vs Less Intensive Hypertension Treatment Regimens Defined by Clinically Noted HDD

Definition of intensive treatment by clinically noted HDDs and pharmacy HDDs	Sensitivity, %	Specificity, %	C statistic (95% CI), %
≥2.0 HDDs	91.5	94.1	92.8 (92.0-93.7)
≥3.0 HDDs	85.7	95.5	90.6 (89.6-91.6)
≥4.0 HDDs	86.1	97.3	91.7 (90.5-92.9)
≥5.0 HDDs	83.3	98.7	91.0 (89.3-92.7)
≥6.0 HDDs	77.2	99.0	88.1 (85.5-90.6)

Abbreviation: HDD, hypertension daily dose.

The distribution of the number of pharmacy fill BP medications (Table 4) demonstrated that, at most visits, veterans were taking 2 medications (1315 visits [36.3%]) or 3 medications (1328 visits [36.6%]). Use of multiple medications was common even at lower HDDs. Nearly one-fourth (76 of 308 [24.7%]) of visits with 0.5 or fewer HDDs were associated with 2 or 3 medications, and 278 of 965 visits (28.8%) with 0.5 to 1.5 HDDs were associated with 3 or 4 medications. Using only the number of medications, 1527 visits (42.1%) would overestimate intensity owing to 1 or more low-dose medications (≤0.5 HDDs). Conversely, the number of medications belied the intensity in 425 visits (11.7%) that include 1 or more medication at maximum beneficial dose or more (≥2 HDDs).

**Table 4.** Association Between Categories of Pharmacy HDD and Number of BP Medication Classes^a^

HDD categories	Visits	No. of BP medication classes							
		0	1	2	3	4	5	6	Mean (SD) [range]
≤0.5	308	67	165^b^	69^b^	7^b^	0	0	0	1.05 (0.73) [0-3]
>0.5 to ≤1.5	965	0	91	596^b^	250^b^	28^b^	0	0	2.22 (0.65) [1-4]
>1.5 to ≤2.5	759	0	61	367	290^b^	40^b^	1^b^	0	2.41 (0.72) [1-5]
>2.5 to ≤3.5	642	0	3^c^	187	394	56^b^	2^b^	0	2.79 (0.61) [1-5]
>3.5 to ≤4.5	441	0	1^c^	53^c^	251	113	23^b^	0	3.24 (0.73) [1-5]
>4.5 to ≤5.5	246	0	0	39^c^	77^c^	107	23	0	3.46 (0.87) [2-5]
>5.5 to ≤6.5	147	0	0	4^c^	46^c^	85^c^	10	2	3.73 (0.69) [2-6]
>6.5	117	0	0	0	13^c^	79^c^	25^c^	0	4.10 (0.56) [3-5]
Total	3625	67	321	1315	1328	508	84	2	2.59 (0.98) [0-6]

Abbreviations: BP, blood pressure; HDD, hypertension daily dose.

^a^ Total of 3625 visits for 319 patients.

^b^ Indicates visits (total 1527 [42.1%]) in which the number of BP medications overestimates intensity owing to 1 or more medications at low dose.

^c^ Indicates visits (total 425 [11.7%]) in which the number of BP medications underestimates intensity owing to 1 or more medications at maximum beneficial dose or more.

## Discussion

We developed a method that standardizes doses across common BP medications based on hypertension trial–proven maximum doses associated with cardiovascular benefit. This method uses pharmacy fill data that are available in health system administrative data and can flexibly be applied to any given day or visit. We validated this new measure—total HDDs, measured on days of primary care visits—and found it to be highly correlated with the dose intensity documented in the clinical notes on those same days.

Previous studies have proposed that measuring treatment intensity for older patients should consider not only whether a BP target is achieved but also the number of medications an older patient is receiving because an older adult with low BP taking 3 medications is being treated more aggressively than an older adult with the same BP but taking no medications or 1 or 2 medications.^13,32^ With this additional dose information, we can gain an even more comprehensive measure of hypertension treatment intensity with far more granularity than with dichotmous BP targets or medication counts alone. In health care systems in which detailed pill counts and patient interviews are not possible, this method may efficiently measure hypertension treatment intensity. Given the many choices of BP medications demonstrated by trials to be associated with clinical benefit, this method offers a continuous measure of treatment intensity that can holistically assess the association between treatment, BP, and outcomes.

In this study, we applied dose information at every primary care visit and validated this measure on those days. This measure can flexibly be applied on any day, for example, at set intervals, such as the last day or last visit of each quarter or year. In an observational study, one could apply the measure at fixed time points after an index day (eg, enrollment into a health plan or after a fixed interval after implementation of a quality improvement intervention).

This method may increase our understanding of prescribing patterns in terms of both HDDs and medication counts in interventional studies. Clinical trials typically describe the intervention effect in terms of medication counts only; adding dose intensity may further convey the intensity of intervention necessary to gain the clinical benefits of the trial. Nonpharmacologic interventions, such as diet and exercise, may also be associated with decreases in hypertension medication intensity as a proximal outcome. For example, one study of improved sleep apnea treatment used reduction in antihypertension medication–adjusted BP as an outcome, thus reflecting improvement in hypertension severity if BP doses were decreased despite an equivalent BP.^33^ An intervention that decreases medication use would be valuable from a health care use perspective and a patient preference perspective. Moreover, because some adverse effects are dose dependent, a decrease in dose would presumably also decrease risk of downstream adverse effects.^10,34,35^

Modern clinical trials have provided evidence of the benefit of treating hypertension using optimized doses of older drugs, such as diuretics and β-blockers, and including newer medications, such as calcium channel blockers, angiotensin-converting enzyme inhibitors, or angiotensin receptor blockers,^15,17,36^ thus allowing us to develop this new taxonomy for standardizing antihypertension doses based on clinical benefit. Our taxonomy improves on the prior ATC/DDD system,^16^ which was intended as a stable measure over time and did not provide “judgements about relative efficacy of drugs and groups of drugs.”^16^ Our new HDD measure may facilitate future study of the trade-offs between multiple low-dose regimens and fewer maximum-dose regimens, benchmarked to trial evidence of cardiovascular benefit.

In this validation sample of older veterans treated for hypertension with multiple medications, we found that a substantial proportion of visits included low doses of those medications. Because guidelines recommend angiotensin-converting enzyme inhibitors or angiotensin receptor blockers for kidney protection for chronic kidney disease and/or diabetes, as well as for cardiovascular prevention for diabetes and atherosclerotic cardiovascular disease,^37,38,39^ we would expect that patients with multiple comorbidities would be taking multiple, but lower-dose, medications to gain benefits for these comorbid conditions.

Furthermore, greater medication regimen complexity has been associated with poorer adherence,^40,41^ but dose burden is understudied as a potential patient-centered factor in BP control. Hypertension daily doses may also be used as a way to help gauge the complexity of patients’ BP medication regimens (eg, as an outcome of a medication simplification intervention).

Despite the very high correlation of the pharmacy HDD with the clinically noted HDD, we found that pharmacy HDDs were lower than clinically noted HDDs. The most likely reason was our method of reducing estimated HDDs for late refills, therefore more closely matching what the patient took. If the patient took fewer pills than recommended (ie, nonadherence), then we would observe a mismatch between pharmacy HDD and clinically noted HDD. Although VA patients report better adherence than non-VA patients owing to lower cost or no cost for filling medications,^42^ our finding is congruent with what has been described for older patients with hypertension.^43^ Last, some of the difference could have resulted from out-of-pocket payments (ie, low-cost generic programs) from filling prescriptions at non-VA pharmacies.

### Strengths and Limitations

This study has some strengths, including the use of 2 sources of pharmacy data (VA system and Medicare Part D), an external source of validation (medical record review), and data from a national health care system. Although there have been many changes to guidelines and BP targets in the last several years, our study preceded these changes (ie, the systolic BP target [<140 mm Hg] was identical to the present).

This study also has several limitations. First, we were unable to validate doses for BP-lowering medications with as-needed or frequent changes (eg, loop diuretics) or for medications infrequently documented for hypertension treatment (eg, α-blockers for prostatic hypertrophy). Second, because our study focused on older veterans, the sample included mostly men, thus potentially limiting generalizability to women, although BP medication fill behavior is similar between the sexes.^44^ Third, this was a cross-sectional validation of dose accuracy. Validation longitudinally for small changes in dose over time, or linked externally to cardiovascular outcomes or adverse events, is beyond the scope of this research. Fourth, as described previously,^13^ we studied a more intensely treated sample of veterans than average, so it is possible that our results may not be generalizable to veterans with less complex treatment regimens.

## Conclusions

We have developed and validated an algorithm to identify the degree of hypertension treatment intensity that is a more precise measure of intensity than number of BP medications or BP alone. Our results may pave the way for further studies of dose intensity and health outcomes, including programs to increase or decrease hypertension treatment intensity or to compare treatment intensity between clinicians, clinics, and health care systems.

## Notes

Supplement. **eTable 1.** Differences Between Anatomic Therapeutic Chemical (ATC)/Daily Defined Dose (DDD) and New Moderate Hypertension Daily Dose (HDD) Measure **eTable 2.** SAS Code for Calculating Intensity of Hypertension Medication Regimens (Class Count and Dose Measured by HDDs) on Any Given Day Using Pharmacy Fill Data

## References

[1] Staessen JA, Gasowski J, Wang JG, Risks of untreated and treated isolated systolic hypertension in the elderly: meta-analysis of outcome trials. Lancet. 2000;355(9207):865-872. doi:10.1016/S0140-6736(99)07330-4 10752701

[2] Wright JT Jr, Williamson JD, Whelton PK, ; SPRINT Research Group A randomized trial of intensive versus standard blood-pressure control. N Engl J Med. 2015;373(22):2103-2116. doi:10.1056/NEJMoa1511939 26551272 PMC4689591

[3] Williamson JD, Supiano MA, Applegate WB, ; SPRINT Research Group Intensive vs standard blood pressure control and cardiovascular disease outcomes in adults aged ≥75 years: a randomized clinical trial. JAMA. 2016;315(24):2673-2682. doi:10.1001/jama.2016.7050 27195814 PMC4988796

[4] Gueyffier F, Bulpitt C, Boissel JP, ; INDANA Group Antihypertensive drugs in very old people: a subgroup meta-analysis of randomised controlled trials. Lancet. 1999;353(9155):793-796. doi:10.1016/S0140-6736(98)08127-6 10459960

[5] Beckett NS, Peters R, Fletcher AE, ; HYVET Study Group Treatment of hypertension in patients 80 years of age or older. N Engl J Med. 2008;358(18):1887-1898. doi:10.1056/NEJMoa0801369 18378519

[6] Mishima E, Haruna Y, Arima H Renin-angiotensin system inhibitors in hypertensive adults with non-diabetic CKD with or without proteinuria: a systematic review and meta-analysis of randomized trials. Hypertens Res. 2019;42(4):469-482. doi:10.1038/s41440-018-0116-3 30948820

[7] Muntner P, Carey RM, Gidding S, Potential US population impact of the 2017 ACC/AHA high blood pressure guideline. Circulation. 2018;137(2):109-118. doi:10.1161/CIRCULATIONAHA.117.032582 29133599 PMC5873602

[8] Centers for Disease Control and Prevention (CDC) Self-reported hypertension and use of antihypertensive medication among adults—United States, 2005-2009. MMWR Morb Mortal Wkly Rep. 2013;62(13):237-244. 23552224 PMC4605009

[9] Gerstein HC, Miller ME, Byington RP, ; Action to Control Cardiovascular Risk in Diabetes Study Group Effects of intensive glucose lowering in type 2 diabetes. N Engl J Med. 2008;358(24):2545-2559. doi:10.1056/NEJMoa0802743 18539917 PMC4551392

[10] Odden MC, Peralta CA, Haan MN, Covinsky KE Rethinking the association of high blood pressure with mortality in elderly adults: the impact of frailty. Arch Intern Med. 2012;172(15):1162-1168. doi:10.1001/archinternmed.2012.2555 22801930 PMC3537835

[11] Tinetti ME, Han L, Lee DS, Antihypertensive medications and serious fall injuries in a nationally representative sample of older adults. JAMA Intern Med. 2014;174(4):588-595. doi:10.1001/jamainternmed.2013.14764 24567036 PMC4136657

[12] Juraschek SP, Miller ER III, Weaver CM, Appel LJ Effects of sodium reduction and the DASH diet in relation to baseline blood pressure. J Am Coll Cardiol. 2017;70(23):2841-2848. doi:10.1016/j.jacc.2017.10.011 29141784 PMC5742671

[13] Min L, Ha JK, Hofer TP, Validation of a health system measure to capture intensive medication treatment of hypertension in the Veterans Health Administration. JAMA Netw Open. 2020;3(7):e205417. doi:10.1001/jamanetworkopen.2020.5417 32729919 PMC9374172

[14] U.S. Department of Veterans Affairs. VA national formulary. Updated December 8, 2020. Accessed May 1, 2015. https://www.pbm.va.gov/nationalformulary.asp

[15] Whelton PK, Carey RM, Aronow WS, 2017 ACC/AHA/AAPA/ABC/ACPM/AGS/APhA/ASH/ASPC/NMA/PCNA guideline for the prevention, detection, evaluation, and management of high blood pressure in adults: a report of the American College of Cardiology/American Heart Association Task Force on Clinical Practice Guidelines. J Am Coll Cardiol. 2018;71(19):e127-e248. doi:10.1016/j.jacc.2017.11.006 29146535

[16] WHO Collaborating Centre for Drug Statistics Methodology. ATC Classification Index with DDD—Excel format, 2017.

[17] Chobanian AV, Bakris GL, Black HR, ; National Heart, Lung, and Blood Institute; National High Blood Pressure Education Program Coordinating Committee Seventh report of the Joint National Committee on Prevention, Detection, Evaluation, and Treatment of High Blood Pressure. Hypertension. 2003;42(6):1206-1252. doi:10.1161/01.HYP.0000107251.49515.c2 14656957

[18] James PA, Oparil S, Carter BL, 2014 Evidence-based guideline for the management of high blood pressure in adults: report from the panel members appointed to the Eighth Joint National Committee (JNC 8). JAMA. 2014;311(5):507-520. doi:10.1001/jama.2013.284427 24352797

[19] Caruso FS, Szabadi RR, Vukovich RA Pharmacokinetics and clinical pharmacology of indapamide. Am Heart J. 1983;106(1, pt 2):212-220. doi:10.1016/0002-8703(83)90119-9 6869203

[20] Chaffman M, Heel RC, Brogden RN, Speight TM, Avery GS Indapamide: a review of its pharmacodynamic properties and therapeutic efficacy in hypertension. Drugs. 1984;28(3):189-235. doi:10.2165/00003495-198428030-00001 6489195

[21] Duchier J, Iannascoli F, Safar M Antihypertensive effect of sustained-release isosorbide dinitrate for isolated systolic systemic hypertension in the elderly. Am J Cardiol. 1987;60(1):99-102. doi:10.1016/0002-9149(87)90993-3 3300248

[22] Kirby RS Doxazosin in benign prostatic hyperplasia: effects on blood pressure and urinary flow in normotensive and hypertensive men. Urology. 1995;46(2):182-186. doi:10.1016/S0090-4295(99)80191-5 7542819

[23] Krum H, Viskoper RJ, Lacourciere Y, Budde M, Charlon V; Bosentan Hypertension Investigators The effect of an endothelin-receptor antagonist, bosentan, on blood pressure in patients with essential hypertension. N Engl J Med. 1998;338(12):784-790. doi:10.1056/NEJM199803193381202 9504938

[24] Levy P Effects of prazosin on blood pressure and diabetic control in patients with type II diabetes mellitus and mild essential hypertension. Am J Med. 1989;86(1B):59-62. doi:10.1016/0002-9343(89)90132-0 2913774

[25] Mondaini N, Giubilei G, Ungar A, Alfuzosin (10 mg) does not affect blood pressure in young healthy men. Eur Urol. 2006;50(6):1292-1296. doi:10.1016/j.eururo.2006.06.016 16837126

[26] Musini VM, Rezapour P, Wright JM, Bassett K, Jauca CD Blood pressure–lowering efficacy of loop diuretics for primary hypertension. Cochrane Database Syst Rev. 2015;(5):CD003825. doi:10.1002/14651858.CD003825.pub4 26000442 PMC7156893

[27] Sundquist H, Anttila M, Arstila M Antihypertensive effects of practolol and sotalol. Clin Pharmacol Ther. 1974;16(3):465-472. doi:10.1002/cpt1974163part1465 4606232

[28] Yoshida M, Kudoh J, Homma Y, Kawabe K Safety and efficacy of silodosin for the treatment of benign prostatic hyperplasia. Clin Interv Aging. 2011;6:161-172. doi:10.2147/CIA.S13803 21753871 PMC3131986

[29] Law MR, Wald NJ, Morris JK, Jordan RE Value of low dose combination treatment with blood pressure lowering drugs: analysis of 354 randomised trials. BMJ. 2003;326(7404):1427. doi:10.1136/bmj.326.7404.1427 12829555 PMC162261

[30] Weintraub WS, Clements SD Jr, Crisco LV, Twenty-year survival after coronary artery surgery: an institutional perspective from Emory University. Circulation. 2003;107(9):1271-1277. doi:10.1161/01.CIR.0000053642.34528.D9 12628947

[31] Steiner JF, Prochazka AV The assessment of refill compliance using pharmacy records: methods, validity, and applications. J Clin Epidemiol. 1997;50(1):105-116. doi:10.1016/S0895-4356(96)00268-5 9048695

[32] Kerr EA, Lucatorto MA, Holleman R, Hogan MM, Klamerus ML, Hofer TP; VA Diabetes Quality Enhancement Research Initiative (QUERI) Workgroup on Clinical Action Measures Monitoring performance for blood pressure management among patients with diabetes mellitus: too much of a good thing? Arch Intern Med. 2012;172(12):938-945. doi:10.1001/archinternmed.2012.2253 22641246 PMC3699173

[33] Bravata DM, Ferguson J, Miech EJ, Diagnosis and treatment of sleep apnea in patients’ homes: the rationale and methods of the “GoToSleep” randomized-controlled trial. J Clin Sleep Med. 2012;8(1):27-35. doi:10.5664/jcsm.1654 22334806 PMC3266333

[34] Gnjidic D, Hilmer SN, Blyth FM, Polypharmacy cutoff and outcomes: five or more medicines were used to identify community-dwelling older men at risk of different adverse outcomes. J Clin Epidemiol. 2012;65(9):989-995. doi:10.1016/j.jclinepi.2012.02.018 22742913

[35] Le Couteur DG, McLachlan AJ, de Cabo R Aging, drugs, and drug metabolism. J Gerontol A Biol Sci Med Sci. 2012;67(2):137-139. doi:10.1093/gerona/glr084 21768500

[36] Chobanian AV, Bakris GL, Black HR, ; National Heart, Lung, and Blood Institute Joint National Committee on Prevention, Detection, Evaluation, and Treatment of High Blood Pressure; National High Blood Pressure Education Program Coordinating Committee The Seventh Report of the Joint National Committee on Prevention, Detection, Evaluation, and Treatment of High Blood Pressure: the JNC 7 report. JAMA. 2003;289(19):2560-2572. doi:10.1001/jama.289.19.2560 12748199

[37] KDIGO Blood Pressure Work Group KDIGO clinical practice guideline for the management of blood pressure in chronic kidney disease. Kidney Int Suppl (2011). 2012;2(5)(suppl):337-414.

[38] Trietley GS, Wilson SA, Chaudhri P, Payette N, Higbea A, Nashelsky J Clinical inquiry: do ACE inhibitors or ARBs help prevent kidney disease in patients with diabetes and normal BP? J Fam Pract. 2017;66(4):257-263. 28375400

[39] American Diabetes Association *Standards of Medical Care in Diabetes–2018* abridged for primary care providers. Clin Diabetes. 2018;36(1):14-37. 29382975 10.2337/cd17-0119 PMC5775000

[40] Ho CP, Yeh JI, Wen SH, Lee TJ Associations among medication regimen complexity, medical specialty, and medication possession ratio in newly diagnosed hypertensive patients: a population-based study. Medicine (Baltimore). 2017;96(45):e8497. doi:10.1097/MD.0000000000008497 29137042 PMC5690735

[41] Osterberg L, Blaschke T Adherence to medication. N Engl J Med. 2005;353(5):487-497. doi:10.1056/NEJMra050100 16079372

[42] Gaffney A, Bor DH, Himmelstein DU, Woolhandler S, McCormick D The effect of Veterans Health Administration coverage on cost-related medication nonadherence. Health Aff (Millwood). 2020;39(1):33-40. doi:10.1377/hlthaff.2019.00481 31905070

[43] Tang KL, Quan H, Rabi DM Measuring medication adherence in patients with incident hypertension: a retrospective cohort study. BMC Health Serv Res. 2017;17(1):135. doi:10.1186/s12913-017-2073-y 28193217 PMC5307770

[44] Krousel-Wood MA, Muntner P, Islam T, Morisky DE, Webber LS Barriers to and determinants of medication adherence in hypertension management: perspective of the cohort study of medication adherence among older adults. Med Clin North Am. 2009;93(3):753-769. doi:10.1016/j.mcna.2009.02.007 19427503 PMC2702217

