# Supplementary material for: A Method to Quantify Mean Hypertension Treatment Daily Dose Intensity Using Health Care System Data
Source: JAMA Netw Open. 2021 Jan 15;4(1):e2034059. doi: 10.1001/jamanetworkopen.2020.34059 (PMC7811181; doi:10.1001/jamanetworkopen.2020.34059)
Supplement: Supplement. — eTable 1. Differences Between Anatomic Therapeutic Chemical (ATC)/Daily Defined Dose (DDD) and New Moderate Hypertension Daily Dose (HDD) Measure eTable 2. SAS Code for Calculating Intensity of Hypertension Medication Regimens (Class Count and Dose Measured by HDDs) on Any Given Day Using Pharmacy Fill Data [file jamanetwopen-e2034059-s001.pdf]

Supplementary Online Content

Min L, Ha JK, Aubert CE, et al. A method to quantify mean hypertension treatment daily dose intensity using health care system data. *JAMA Netw Open.* 2021;4(1):e2034059. doi:10.1001/jamanetworkopen.2020.34059

eTable 1. Differences Between Anatomic Therapeutic Chemical (ATC)/Daily Defined Dose (DDD) and New Moderate Hypertension Daily Dose (HDD) Measure
eTable 2. SAS Code for Calculating Intensity of Hypertension Medication Regimens (Class Count and Dose Measured by HDDs) on Any Given Day Using Pharmacy Fill Data

This supplementary material has been provided by the authors to give readers additional information about their work.

© 2021 Min L et al. *JAMA Network Open.*

eTable 1. Differences Between Anatomic Therapeutic Chemical (ATC)/Daily Defined Dose (DDD)[16] and New Moderate Hypertension Daily Dose (HDD) Measure

Class	Agent	DDD	HDD	Difference	
BB	Labetalol	600	400	-200	DDD greater than HDD
BB	Nadolol	160	80	-80	
BB	Propranolol	160	80	-80	
BB	Metoprolol	150	100	-50	
Potassium sparing diuretic	Triamterene	100	50	-50	
BB	Atenolol	75	50	-25	
Potassium sparing diuretic	Spironolactone	75	50	-25	
BB	Penbutolol	40	20	-20	
BB	Carvedilol	37.5	25	-12.5	
Diuretic	Chlorthalidone	25	12.5	-12.5	
BB	Bisoprolol	10	5	-5	
Potassium sparing diuretic	Amiloride	10	5	-5	
CCB	Nisoldipine	20	17	-3	
Sympathetic blockers	Guanfacine	3	1	-2	
Sympathetic blockers	Reserpine	0.5	0.125	-0.375	
Sympathetic blockers	Clonidine (patch)	0.45	0.2	-0.25	
Sympathetic blockers	Clonidine	0.45	0.4	-0.05	
ACEI	Captopril	50	50	0	HDD equal to DDD
ACEI	Moexipril	15	15	0	
ACEI	Trandolapril	2	2	0	
BB	Acebutolol	400	400	0	
CCB	Amlodipine	5	5	0	
CCB	Diltiazem	240	240	0	
CCB	Felodipine	5	5	0	
CCB	Isradipine	5	5	0	
CCB	Nicardipine	90	90	0	
CCB	Verapamil	240	240	0	
Smooth muscle vasodilator	Hydralazine	100	100	0	
ARB	Azilsartan	40	40	0	
ARB	Eprosartan	600	600	0	
ARB	Irbesartan	150	150	0	
ARB	Losartan	50	50	0	
ARB	Olmesartan	20	20	0	
ARB	Telmisartan	40	40	0	
Diuretic	Hydrochlorothiazide	25	25	0	
Diuretic	Indapamide	2.5	2.5	0	
Diuretic	Metolazone	5	5	0	
Potassium sparing diuretic	Eplerenone	50	50	0	
Arteriole dilator	Minoxidil	20	20	0	

© 2021 Min L et al. *JAMA Network Open.*

Direct Renin inhibitors	Aliskiren	150	150	0	
Diuretic	Polythiazide	1	2	1	DDD less than HDD
Diuretic	Bendroflumethiazide	2.5	5	2.5	
ACEI	Perindopril	4	8	4	
ACEI	Fosinopril	15	20	5	
ACEI	Ramipril	2.5	10	7.5	
ARB	Candesartan	8	16	8	
ACEI	Enalapril	10	20	10	
ACEI	Lisinopril	10	20	10	
ACEI	Benazepril	7.5	20	12.5	
BB	Nebivolol	5	20	15	
BB	Pindolol	15	30	15	
ACEI	Quinapril	15	40	25	
CCB	Nifedipine	30	60	30	
ARB	Valsartan	80	160	80	
Sympathetic blockers	Methyldopa	200	500	300	

BB = Beta blockers

ARB = Angiotensin receptor blocker

ACEI = angiotensin converting enzyme inhibitor

CCB = calcium channel blocker

© 2021 Min L et al. *JAMA Network Open.*

eTable 2. SAS Code for Calculating Intensity of Hypertension Medication Regimens (Class Count and Dose Measured by HDDs) on Any Given Day Using Pharmacy Fill Data

/***

[Visits] is encounter file which has

- Patient ID (**PatientID**)
- Encounter Date (**VisitDate**)

[Rx] is medication file which has

- Patient ID (**PatientID**)
- Release Date (**ReleaseDate**)
- Drug name without dose (**DNWD**)
- Days of Supply (**DaysSupply**)
- **PRN** indicator: 1 if it's PRN medication
- **Strength** of a medicine (mg)
- Quantity given (**QtyNumeric**)
- Medication routes: **MedRoute** = 'O' (for oral) or 'PATCH' (for patch)

[HDD table] from Table 1

- Medication name (**Medication**)
- Medication routes: **MedRoute** = 'O' (for oral) or 'PATCH' (for patch)
- Geriatric starting dose (**min**)
- Dose corresponding to one HDD (**med**)
- Maximum dose based on reference (**max**)

***/

```
/* Define class (AntiHTNclass) and NewRx_ID which is used to identify new and old medications ****/
Data [Rx];
 set [Rx];
if DNWD in ('AZILSARTAN', 'BENAZEPRIL', 'CANDESARTAN', 'CAPTOPRIL', 'ENALAPRIL','EPROSARTAN',
                 'FOSINOPRIL', 'IRBESARTAN', 'LISINOPRIL', 'LOSARTAN', 'MOEXIPRIL', 'OLMESARTAN',
                 'PERINDOPRIL', 'QUINAPRIL', 'RAMIPRIL', 'TELMISARTAN', 'TRANDOLAPRIL', 'VALSARTAN')
                  then AntiHTNclass = 1;

else if DNWD in ('AMLODIPINE', 'DILTIAZEM', 'FELODIPINE', 'ISRADIPINE', 'NICARDIPINE', 'NIFEDIPINE',
                     'NISOLDIPINE', 'VERAPAMIL') then AntiHTNclass = 2;

else if DNWD in ('BENDROFLUMETHIAZIDE', 'CHLORTHALIDONE', 'HYDROCHLOROTHIAZIDE',
                     'INDAPAMIDE', 'METOLAZONE', 'POLYTHIAZIDE')
                      then AntiHTNclass = 3;

else if DNWD in ('AMILORIDE','EPLERENONE','SPIRONOLACTONE','TRIAMTERENE')
                       then AntiHTNclass = 4;

else if DNWD in ('ACEBUTOLOL', 'ATENOLOL', 'BISOPROLOL', 'CARVEDILOL', 'LABETALOL',
                       'METOPROLOL', 'NADOLOL', 'NEBIVOLOL', 'PENBUTOLOL', 'PINDOLOL',
'PROPRANOLOL',
                       'SOTALOL') then AntiHTNclass = 5;

else if DNWD in ('CLONIDINE', 'GUANFACINE', 'METHYLDOPA','RESERPINE') then AntiHTNclass = 6;

else if DNWD in ('HYDRALAZINE', 'MINOXIDIL') then AntiHTNclass = 7;

else if DNWD in ('ALISKIREN') then AntiHTNclass = 8;

else if DNWD in ('DOXAZOSIN', 'PRAZOSIN', 'SILODOSIN', 'TERAZOSIN') then AntiHTNclass =  9;
```

© 2021 Min L et al. *JAMA Network Open.*

```
else if DNWD in ('BUMETANIDE', 'FUROSEMIDE', 'TORSEMIDE') then AntiHTNclass = 10;

else if DNWD in ('ISOSORBIDE DINITRATE', 'ISOSORBIDE MONONITRATE') then AntiHTNclass = 11;
else AntiHTNclass = .;

 NewRx_ID = DNWD;
 if AntiHTNclass = 1 then do;
    if DNWD in ('BENAZEPRIL', 'CAPTOPRIL', 'ENALAPRIL', 'FOSINOPRIL', 'LISINOPRIL', 'MOEXIPRIL',
                  'PERINDOPRIL', 'QUINAPRIL', 'RAMIPRIL', 'TRANDOLAPRIL') then NewRx_ID = 'Class_1A';
    else NewRx_ID = 'Class_1B'; end;
run;


/* Choose the last release date of medication before the date of interest  (e.g., we used visit date but it can be
any index date) ***************************************************************/
proc sql;
 create table Last_ReleaseDate as
 select a.PatientID, a.VisitDate, b.AntiHTNclass, b.DNWD, b.NewRx_ID,
          max(b.ReleaseDate) as Last_ReleaseDate format = date9.
 from [Visits] as a
 left join [Rx] as b on (a.PatientID = b.PatientID)
 where intnx('day', a.VisitDate, -186) <= b.ReleaseDate and b.ReleaseDate < a.VisitDate
group by 1,2,3,4,5,6;
quit;

proc sql;
 create table Pre as
 select a.*, b.DaysSupply, b.PRN, b.Strength, b.QtyNumeric, b.MedRoute
 from Last_ReleaseDate as a
 left join [Rx] as b on (a.PatientID = b.PatientID and a.DNWD = b.DNWD and
                             a.Last_ReleaseDate = b.ReleaseDate)
quit;

/* To decide whether the medication is newly-prescribed or an old medication ********************/
proc sql;
 create table NewRx0 as
 select a.PatientID, a.VisitDate, a.DNWD, a.NewRx_ID,
          max(a.Last_ReleaseDate) as Last_ReleaseDate format = date9.
 from Last_ReleaseDate as a
 group by 1,2,3,4;
quit;
proc sql;
 create table NewRx as
 select a.PatientID, a.VisitDate, a.DNWD, a.NewRx_ID, a.Last_ReleaseDate,
         count(case when b.ReleaseDate ne . then b.ReleaseDate end) as N_PreRx
 from NewRx0 as a
 left join [Rx] as b on (a.PatientID = b.PatientID and a.NewRx_ID = b.NewRx_ID and
                               intnx('year', a.Last_ReleaseDate, -2) <= b.ReleaseDate and
                               b.ReleaseDate < a.Last_ReleaseDate)
 group by 1,2,3,4,5;
quit;

proc sql;
 create table Pre1 as
 select a.*,
```

© 2021 Min L et al. *JAMA Network Open.*

```
           case when b.N_PreRx = 0 then 1 else 0 end as NewRx
 from Pre as a
 left join NewRx as b on (a.PatientID = b.PatientID and a.VisitDate = b.VisitDate and a.DNWD = b.DNWD
                                   and a.NewRx_ID = b.NewRx_ID);
quit;


/* Define the potential end date for a discontinued medication**********************************/
proc sql;
 create table PreRx_combine as
 select *,
           case when NewRx = 1 then intnx('day', Last_ReleaseDate, DaysSupply*0.8)
                 when NewRx = 0 then intnx('day', Last_ReleaseDate, DaysSupply*0.9)
                 else . end as Potential_EndDate format = date9.,

           case when NewRx = 1 and intnx('day', Last_ReleaseDate, DaysSupply*0.8) >= VisitDate then 1
                 when NewRx = 0 and intnx('day', Last_ReleaseDate, DaysSupply*0.9) >= VisitDate then 1
                 else 0 end as StillOn
 from Pre1;
quit;

/* To check if the medication is refilled ****************************************************/
/* 1. Non-PRN or PRN medication is refilled by the same class medication */
proc sql;
 create table Refilled_byClass as
 select a.PatientID, a.VisitDate, a.AntiHTNclass, a.DNWD, a.PRN, a.Last_ReleaseDate,
           min(b.ReleaseDate) as RefillDate_byClass format date9.
 from PreRx_combine as a
 left join [Rx] as b on (a.PatientID = b.PatientID)
 where a.PRN ne 1 and a.VisitDate <= b.ReleaseDate and b.ReleaseDate <= intnx('day', a.VisitDate, 186)
            and a.AntiHTNclass = b.AntiHTNclass
 group by 1,2,3,4,5,6
union all
 select a.PatientID, a.VisitDate, a.AntiHTNclass, a.DNWD, a.PRN, a.Last_ReleaseDate,
           min(b.ReleaseDate) as RefillDate_byClass format date9.
 from PreRx_combine as a
 left join [Rx] as b on (a.PatientID = b.PatientID)
 where a.PRN = 1 and a.VisitDate <= b.ReleaseDate and b.ReleaseDate < intnx('year', a.VisitDate, 1) and
            a.AntiHTNclass = b.AntiHTNclass
 group by 1,2,3,4,5,6;
quit;

/* 2. Non-PRN medication is refilled by the same medication */
proc sql;
 create table Refilled_byDNWD_NonPRN as
 select a.PatientID, a.VisitDate, a.AntiHTNclass, a.DNWD, a.PRN, a.Last_ReleaseDate,
           min(c.ReleaseDate) as RefillDate_byDNWD format date9.
 from PreRx_combine as a
 left join [Rx] as c on (a.PatientID = c.PatientID)
 where a.PRN ne 1 and a.VisitDate <= c.ReleaseDate and c.ReleaseDate < intnx('day', a.VisitDate, 186)
           and a.DNWD = c.DNWD
 group by 1,2,3,4,5,6;
quit;

/* 3. PRN medication is refilled by the same medication:
    If it’s refilled by both PRN and non-PRN medication, consider the release date of PRN as refill date */
```

© 2021 Min L et al. *JAMA Network Open.*

```
proc sql;
 create table Refilled_byDNWD_PRN0 as
 select a.PatientID, a.VisitDate, a.AntiHTNclass, a.DNWD, a.PRN, a.Last_ReleaseDate,
           min(c.ReleaseDate) as RefillDate_byDNWD_PRN0 format date9.
 from PreRx_combine as a
 left join [Rx] as c on (a.PatientID = c.PatientID)
 where a.PRN = 1 and a.VisitDate <= c.ReleaseDate and c.ReleaseDate < intnx('year', a.VisitDate, 1) and
            a.DNWD = c.DNWD
 group by 1,2,3,4,5,6;
quit;
proc sql;
 create table Refilled_byDNWD_PRN1 as
 select a.PatientID, a.VisitDate, a.AntiHTNclass, a.DNWD, a.PRN, a.Last_ReleaseDate,
           min(c.ReleaseDate) as RefillDate_byDNWD_PRN1 format date9.
 from PreRx_combine as a
 left join [Rx] as c on (a.PatientID = c.PatientID)
 where a.PRN = 1 and a.VisitDate <= c.ReleaseDate and c.ReleaseDate < intnx('year', a.VisitDate, 1) and
            a.DNWD = c.DNWD and c.PRN = 1
 group by 1,2,3,4,5,6;
quit;
proc sql;
 create table Refilled_byDNWD_PRN as
 select a.PatientID, a.VisitDate, a.AntiHTNclass, a.DNWD, a.PRN, a.Last_ReleaseDate,
          case when b.RefillDate_byDNWD_PRN1 is not NULL then b.RefillDate_byDNWD_PRN1
                when a.RefillDate_byDNWD_PRN0 is not NULL then a.RefillDate_byDNWD_PRN0
                 else . end as RefillDate_byDNWD format date9.
 from Refilled_byDNWD_PRN0 as a
 left join Refilled_byDNWD_PRN1 as b on (a.PatientID = b.PatientID and a.VisitDate = b.VisitDate and
                                                          a.DNWD = b.DNWD and a.PRN = b.PRN and
                                                         a.Last_ReleaseDate = b.Last_ReleaseDate);
quit;

proc sql;
 create table Refilled_byDNWD as
 select *
 from Refilled_byDNWD_NonPRN
union all
 select *
 from Refilled_byDNWD_PRN;
quit;

/* If it’s a continuous med, identify the refill date by the same med or same class of med.
    If it’s a discontinued med, take the potential end date as the refill date. *********************/
proc sql;
 create table Rx1 as
 select a.*, b.RefillDate_byDNWD, d.RefillDate_byClass format date9.,
           case when b.RefillDate_byDNWD is not NULL then b.RefillDate_byDNWD
                   when d.RefillDate_byClass is not NULL then d.RefillDate_byClass
                  when a.StillOn = 1 then a.Potential_EndDate
                  else . end as RefillDate format date9.
 from PreRx_combine as a
 left join Refilled_byClass as d on (a.PatientID = d.PatientID and a.VisitDate = d.VisitDate and
                                                 a.DNWD = d.DNWD and a.Last_ReleaseDate = d.Last_ReleaseDate and
                                                 a.PRN = d.PRN)
 left join Refilled_byDNWD as b on (a.PatientID = b.PatientID and a.VisitDate = b.VisitDate and
                                                  a.DNWD = b.DNWD and a.Last_ReleaseDate = b.Last_ReleaseDat and
```

© 2021 Min L et al. *JAMA Network Open.*

```
                                        a.PRN = b.PRN);
quit;

/* Drop possible double counts:
    If medication A and B in the same class but only A is refilled,
    then consider only A. B is not refilled ***************************************************/
proc sql;
 create table Double_Count as
 select PatientID, VisitDate, AntiHTNClass,
          count(case when RefillDate_byDNWD is not NULL then RefillDate_byDNWD end) as N_byDNWD
 from Rx1
 group by 1,2,3;
quit;

/* Define the refill date */
proc sql;
 create table RefillDate as
 select a.PatientID, a.VisitDate, a.Last_ReleaseDate,
          a.AntiHTNclass, a.DNWD, a.PRN, a.DaysSupply, a.Strengh, a.QtyNumeric,
          case when a.RefillDate_byDNWD = . and b.N_byDNWD > 0 then .
          else a.RefillDate end as RefillDate format date9.
 from Rx1 as a
 left join Double_Count as b on (a.PatientID = b.PatientID and a.VisitDate = b.VisitDate and
                                        a.AntiHTNClass = b.AntiHTNClass);
quit;

/* Define daily dose and HDD ***********************************************************/
/* For medications that are refilled later than the prior day's supply, the daily dose is adjusted downward
proportionally (Adj) */
proc sql;
 create table DD as
 select a.*,
          a.Strength*a.QtyNumeric/a.DaysSupply as DailyDose0,
          case when a.RefillDate = . then .
                when a.PRN ne 1 and a.Last_ReleaseDate + a.DaysSupply >= a.VisitDate then 1
                when a.PRN ne 1 and a.Last_ReleaseDate + a.DaysSupply < a.VisitDate then
a.DaysSupply/(a.RefillDate - a.Last_ReleaseDate)
                when a.PRN = 1 and a.DaysSupply >= (a.RefillDate - a.Last_ReleaseDate) then 1
                when a.PRN = 1 and a.DaysSupply < (a.RefillDate - a.Last_ReleaseDate)  then
a.DaysSupply/(a.RefillDate - a.Last_ReleaseDate)
                else . end as Adj,
          b.med, b.max
 from RefillDate as a
 left join [HDD table] as b on (a.DNWD = b.Medication and a.MedRoute = b.MedRoute);
quit;

data FinalData;
 set DD;

 DailyDose = DailyDose0 * Adj;



  /* Other cleaning/programming notes.  We found in the field for days supply for clonidine patches was number
of patches which was a 7 day supply, so the following code corrects for this error ***
  if MedRoute = 'PATCH' then DailyDose = DailyDose0 * 7 * Adj;
```

© 2021 Min L et al. *JAMA Network Open.*

```
  */

  /* Quick fix code in large administrative datasets (not this validation study). In administrative data, many HDD
errors occur when the days' supply in CPRS erroneously low (i.e., 90 pills for 1-day supply instead of 90 days,
resulting in HDD of 90 instead of 1; or 90 pills for a 30-day supply instead of 90 days, resulting in HDD that is 3
times higher than it should be).  Flagging and correcting the outliers might be necessary depending on the
study design.  HDDs can be painstakingly hand corrected by reviewing the instructions variable. This is very
time-consuming.  For a cross sectional analysis, one shortcut that quickly identifies HDDs that are more than 3
times the maximum beneficial dose (~ 6 HDDs for most medications) replaces the HDD at maximum of 6
HDDs.
*/

  HDD_med = DailyDose/med;
  if HDD_med <= 6 then HDD = HDD_med;
  else if HDD_med > 6 then HDD = 6;
  */
run;

/* In longitudinal studies, another option is to flag erroneous HDDs that are >3 times larger or smaller than in
the prior time period (not shown) and either (1) hand-reviewing the instructions variable, (2) imputing the prior
dose especially if the HDD returns to baseline in the next time period or (3) dropping the spurious observation.
*/
```

© 2021 Min L et al. *JAMA Network Open.*
